# Independent component analysis algorithms for non-invasive fetal electrocardiography

**DOI:** 10.1371/journal.pone.0286858

**Published:** 2023-06-06

**Authors:** Rene Jaros, Katerina Barnova, Radana Vilimkova Kahankova, Jan Pelisek, Martina Litschmannova, Radek Martinek

**Affiliations:** 1 Department of Cybernetics and Biomedical Engineering, Faculty of Electrical Engineering and Computer Science, VSB–Technical University of Ostrava, Ostrava, Czechia; 2 Department of Applied Mathematics, Faculty of Electrical Engineering and Computer Science, VSB–Technical University of Ostrava, Ostrava, Czechia; Islamia University of Bahawalpur: The Islamia University of Bahawalpur Pakistan, PAKISTAN

## Abstract

The independent component analysis (ICA) based methods are among the most prevalent techniques used for non-invasive fetal electrocardiogram (NI-fECG) processing. Often, these methods are combined with other methods, such adaptive algorithms. However, there are many variants of the ICA methods and it is not clear which one is the most suitable for this task. The goal of this study is to test and objectively evaluate 11 variants of ICA methods combined with an adaptive fast transversal filter (FTF) for the purpose of extracting the NI-fECG. The methods were tested on two datasets, Labour dataset and Pregnancy dataset, which contained real records obtained during clinical practice. The efficiency of the methods was evaluated from the perspective of determining the accuracy of detection of QRS complexes through the parameters of accuracy (ACC), sensitivity (SE), positive predictive value (PPV), and harmonic mean between SE and PPV (F1). The best results were achieved with a combination of FastICA and FTF, which yielded mean values of ACC = 83.72%, SE = 92.13%, PPV = 90.16%, and F1 = 91.14%. Time of calculation was also taken into consideration in the methods. Although FastICA was ranked to be the sixth fastest with its mean computation time of 0.452 s, it had the best ratio of performance and speed. The combination of FastICA and adaptive FTF filter turned out to be very promising. In addition, such device would require signals acquired from the abdominal area only; no need to acquire reference signal from the mother’s chest.

## Introduction

Fetal electrocardiography (fECG) is a method based on sensing electrical activity of fetal heart in the form of electric potentials. The fECG recording provides clinically important information about the fetus’ medical condition and can be used timely to identify congenital heart defects (such as fetal arrhythmia or fetal atrioventricular block), but most importantly to timely identify fetal hypoxia [1, 2].

Reduced oxygen and blood supply to fetal brain can cause hypoxic-ischemic encephalopathy (HIE), resulting in long-term neurological disorders like mental impairment and cerebral palsy [3]. Accurate monitoring of fetal oxygen and blood supply is crucial to prevent these healthcare concerns. Diagnostic tools are continuously being developed for fetal surveillance. The most used methods are fetal heart rate (fHR) monitoring, fetal scalp blood sampling, ultrasound, and magnetic resonance imaging [4]. Fetal heart rate monitoring is particularly important during the labor. Currently, cardiotocography (CTG) is the most prevalent method for non-invasive electronic monitoring worldwide, as it allows concurrent monitoring of fetal heart rate (fHR) and uterine contractions [5]. However, it has several disadvantages, including a high rate of false positive results, which may lead to unnecessary interventions such as emergency caesarean sections [6, 7]. Therefore, the current research efforts focus on finding alternative methods for non-invasive fHR monitoring, which would be able to detect fetal hypoxia with higher reliability.

One of these methods is fECG, which is able to provide additional information about the fetal health state besides fHR, which is determined using the most prominent peaks of the ECG waveform, the R waves [8]. With fECG, it is possible to accurately identify adverse events during labor (hypoxic conditions) manifested by morphological changes to the fECG signal, particularly ST segment [9]. It can also help in determining other haphazard states during the pregnancy based on the changes of other parts of the waveform, such as QT interval [10–12].

Monitoring with fECG can be done by invasive sensing with a scalp electrode or by non-invasive sensing on the surface of the mother’s abdomen. The invasive variant yields fECG signal with very high quality, there is however a risk of introducing an infection into the uterus, and the variant as such may be carried out only during delivery, after the rupture of membranes. The non-invasive variant is much safer compared to the invasive fECG and may be used both during pregnancy and during delivery [1, 5]. Moreover, unlike CTG, it allows a truly long-term continuous monitoring, as the method is completely passive and neither the mother nor the fetus are exposed to ultrasound energy. Also, electric signals coming from the uterus during contractions can be acquired in addition to the fECG signal. The advantage of non-invasive fECG (NI-fECG) is the possibility to incorporate it into a device for continual remote home monitoring (fetal Holter), particularly useful in high-risk pregnancies or in suspected arrhythmias [13]. This would allow the doctor to remotely monitor the medical condition of the mother as well as of the fetus in real time and the number of actual visits to the doctor could be reduced [14]. Another advantage of NI-fECG is the ease of use and the fact that its quality does not depend much on the operator’s experience.

The disadvantage of the non-invasive variant is that the ECG acquired through abdomen (aECG) is a mix of useful signals, fECG, maternal ECG (mECG), and noise (motion artifacts, myopotentials, isoelectric line fluctuations) [1], see Eq (1), where *n* denotes the number of samples of each vector.

$$\begin{array}{c} \vec{aECG}\left(n\right)=\vec{fECG}\left(n\right)+\vec{mECG}\left(n\right)+\vec{noise}\left(n\right). \end{array}$$ (1)

In addition, the mECG amplitude is usually higher than the fECG amplitude and the mECG signal has almost identical range from the perspective of time and frequency as the fECG and its elimination requires advanced extraction algorithms or combinations thereof [15, 16].

The following section includes a review of the state-of-the-art methods for fECG extraction, with particular focus on the *hybrid* systems. The aim of this article is to identify and test the most promising methods for fECG extraction in terms of the performance, implementability and computational speed.

## State-of-the-art fECG extraction methods

Various algorithms for fECG extraction were tested in the past, among which are techniques based on blind source separation (BSS) [17], wavelet transform (WT) [18], empirical mode decomposition (EMD) [19, 20], template subtraction [21] or adaptive algorithms [15, 16]. The most promising results for fECG extractions acquired in our prior studies [15, 16] were achieved with adaptive algorithms, which used aECG and mECG signal inputs. The mECG signal was modified by the adaptive algorithm so that it corresponds to the maternal component of the aECG signal as much as possible. This modified mECG could be then subtracted from the aECG to acquire the fECG signal as a result.

In general, there are two approaches to acquiring aECG and mECG signal in a non-invasive manner. The first approach is based on sensing aECG signal from abdominal area of the mother, which is used as the primary input, while a reference mECG signal is acquired from the mother’s chest area. However, the mECG signal acquired from the chest has different morphology than the maternal component of the aECG signal and is usually of poor quality and the extraction using the adaptive algorithm is therefore less effective. The measurement process is also less comfortable for the mother and reduces her mobility.

**Fig 1:**
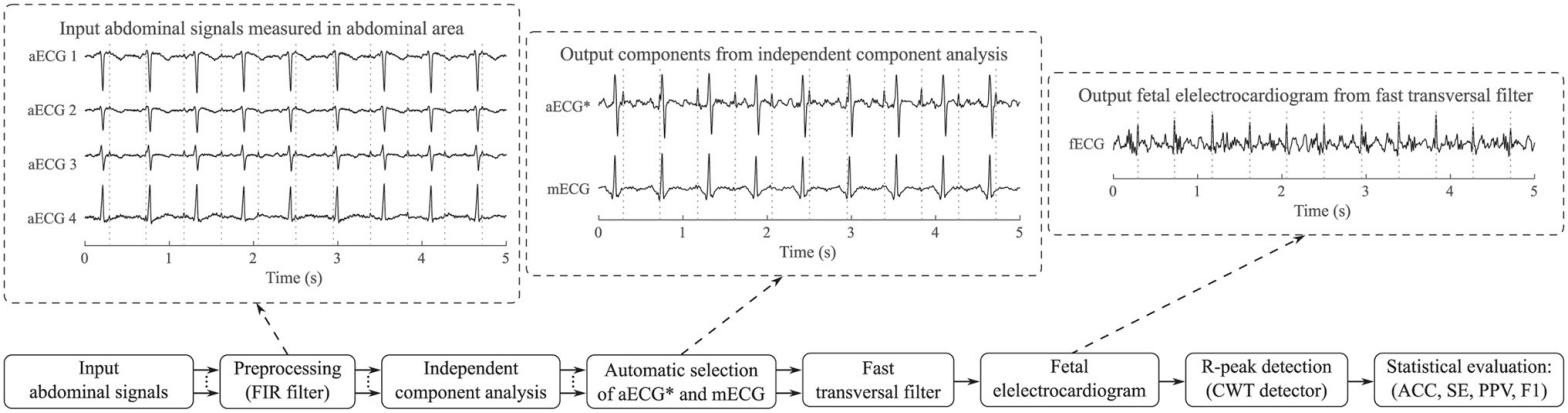
Block diagram illustrating the principle of our hybrid system with examples of the signals in individual phases.

Therefore, we used the second approach to acquire the aECG and mECG input signals. It is based on sensing aECG signal only and on BSS algorithm, which is able to reliably estimate mECG and aECG signal with enhanced fetal component (marked aECG*) from a mix of abdominal signals [15, 16]. Both extracted signals can be used as an input signal for adaptive algorithm, which is then used to modify the mECG signal according to the maternal component in the aECG* signal. The modified signal is then subtracted from aECG*, thus creating a resulting fECG signal (example of aECG input signals, aECG* and mECG components estimated by the ICA-based method and extracted fECG signals is shown in Fig 1).

Since the performance of the adaptive algorithm depends on the quality of input signals, selecting a suitable BSS algorithm is important and has a direct effect on the quality of the estimated aECG* and mECG. Most BSS methods use second- and higher-order statistics, while the source signals and the mixing process are unknown. There are various BSS-based approaches which were tested in the past for fECG extraction. Among those are the most commonly used independent component analysis (ICA) [17, 22–25], principal component analysis (PCA) [22, 25], or non-negative matrix factorization (NMF) [2, 19].

- A comparison of effectiveness of the kurtosis-based FastICA method, negentropy-based FastICA, joint approximate diagonalization of eigenmatrix (JADE) algorithm and PCA for extracting fECG were presented in [22]. The results were evaluated objectively by using the signal-to-noise-ratio (SNR) parameter and subjectively by comparing the waveforms of extracted signals. According to the authors, the best results were achieved with JADE, but the FastICA method proved effective from the perspective of computation time.
- Periodic component analysis has been designed and tested for fECG extraction in [26]. No statistical results were presented, and the extracted signals were merely subjectively evaluated. However, the authors believe that the method was effective from the perspective of both, performance and time. In addition, the assumption of periodic component analysis based on the aperiodic temporal structure criterion was more reasonable in connection with ECG than the assumption of the conventional ICA based on the independence criterion.
- In [23], the authors designed and tested non-parametric ICA based on the kernel density estimation method. The method was tested on synthetic records and the extraction quality was evaluated by means of SNR parameter. Non-parametric ICA managed to extract the fECG even in those records where FastICA and JADE methods failed. The disadvantage of this method is increased computational complexity.
- A comparison of ICA-based methods: second-order blind identification (SOBI), algorithm for extraction of multiple unknown sources (AMUSE) and eigenvalue decomposition was carried out in [24]. Real records were used to test the methods and the results were compared using the sensitivity (SE) and positive predictive value (PPV) parameters. The best results of fECG extraction were achieved with SOBI method (SE = 75.1% and PPV = 69.7% in off-line extraction and SE = 59% and PPV = 46% during on-line extraction).
- A combination of ICA and PCA method was tested on real records in [25]. The ICA method turned out to be effective for the enhancement of fECG and the PCA method managed to effectively suppress mECG. However, the authors recommended validation of this method on signals from women in earlier stages of pregnancy.
- The NMF method was designed for fECG extraction in [2] and tested on real records. Its performance was then compared to the ICA. The methods were tested directly on the original aECG input signals, on aECG signals in compressed domains, and in the recovered aECG after compression. The quality of extraction was evaluated using SE, PPV, and F1 parameters. Based on the F1 parameter, the NMF method proved to be a better solution when used for original aECG signals (NMF yielded an mean of F1 = 94.8%, while ICA yielded F1 = 93.6%) and for recovered aECG (NMF yielded an mean of F1 = 96.75%, while ICA yielded F1 = 95%). On the other hand, ICA turned out to be more suitable for signals in compressed domain (ICA yielded an mean of F1 = 92.5%, while NMF yielded F1 = 24%).
- NMF was tested in combination with the EMD method by the authors in [19]. The EMD method was applied on the aECG input signal, which decomposed it into multiple intrinsic mode functions. A non-negative matrix was created based on the extracted intrinsic mode functions and the number of estimated independent components, to which NMF was then applied and separated mECG and fECG. The method was tested on real and synthetic records and evaluated based on SNR and visual comparison of the extracted signals. With the help of the designed EMD-NMF method, higher SNR and more effective fECG extraction were achieved compared to the single channel ICA and combination of WT-ICA.

Based on the conducted review of the state-of-the-art BSS methods used in fetal ECG extraction, the ICA-based methods appeared to be the most prevalent and also most promising for NI-fECG processing, at least as one of its steps. Often, these algorithms are combined with other methods, such adaptive algorithms [15, 16]. However, there are many variants of the ICA method (FastICA, JADE, SOBI, AMUSE, etc.). Therefore, before implementing newer techniques, it is necessary to examine these conventional methods in detail and determine their strengths and weaknesses so that the new methods can be compared with the conventional ones and see whether their application provides improvement or not. Currently, there is no extensive objective comparison of various ICA-based methods in the field of NI-fECG, and it is therefore not clear which of the ICA algorithms is the most suitable for this task.

The goal of this study is therefore to test and objectively evaluate eleven ICA methods in terms of their performance and computational speed. The tests will include following ICA variants: AMUSE algorithm, equivariant robust ICA algorithm (ERICA), FastICA algorithm, flexible ICA algorithm (FlexICA), logistic infomax ICA algorithm (Infomax), JADE, kernel ICA algorithm (KICA), robust accurate direct ICA algorithm (RADICAL), robust ICA algorithm (RobustICA), simultaneous blind signal extraction using cumulants (SIMBEC), SOBI algorithm.

## Material and methods

This chapter provides basic description of the tested ICA-based method and adaptive FTF algorithm. The real datasets used for objective evaluation and validation of function of the applied algorithms will be described later in this chapter.

Fig 1 shows in block diagram an example of the extraction procedure for recording r9 of the Pregnancy dataset to illustrate our experiments. In this figure, we provide examples of preprocessed input aECG signals by finite impulse response (FIR) filter. Second example shows aECG* and mECG components estimated by the ICA method. In the aECG* signal, the fetal component is enhanced and and thus more prominent. Third example then represents the resulting fECG signal, obtained after modification of the mECG signal with FTF algorithm and subtraction of the modified mECG signal from the aECG* signal.

### Independent component analysis based methods

Independent component analysis is a method that aims to find linear representation of non-Gaussian data so that the components are statistically independent or as independent as possible. Such representation captures the basic structure of data in many applications, including the extraction of functions and signal separation [27].

Before applying the ICA method to aECG input signals, it is often wise to pre-process the signals by centering and whitening. After centering, input signals have zero mean value, whereas whitening makes them discorrelated with the unit variance [27].

For basic motivation, consider a signal sensed from an abdominal area of a pregnant woman (aECG) with multiple signal sources, such as fECG, mECG, electrical activity of the uterus and other muscles (manifesting as electrohysterogram (EHG) and electromyogram (EMG), respectively) and noise caused by the ambient environment. For further description, these sources are defined as $\vec{s}$ and their acquisition from the abdominal area creates mixed signals $\vec{x}$, where each source needs to be separated. Standard linear model of the ICA method can be described by Eq (2), where $\vec{x}$ represents a mix of signals consisting of mutually independent sources $\vec{s}$. The number of signals (measuring electrodes) $\vec{x}$ must not be lower than the number of source signals $\vec{s}$. Mixing matrix **A** is unknown; therefore, the goal of ICA method is to estimate the inverse of demixing matrix **W**. Eq (3) describes the estimation of output independent components $\vec{y}$ using the estimated demixing matrix **W**. Each ICA-based method differs mainly in the manner of making the matrix estimate [27].

$$\begin{array}{c} \vec{x}\left(t\right)=A\cdot \vec{s}\left(t\right). \end{array}$$ (2)

$$\begin{array}{c} \vec{y}\left(t\right)=W\cdot \vec{x}\left(t\right). \end{array}$$ (3)

There are many different ICA-based methods. In this study, the methods described below were used for the experiment (official versions of each algorithm provided by the creators of the algorithms were used to increase the reproducibility of the experiments):

- *AMUSE algorithm* is using eigenvalue value decomposition. This is an algorithm of blind separation of second-order sources, which uses the structure in data to find non-correlating components. To do so, it decomposes singular values on a shifted cross-covariance matrix. The shift is selected so that the auto-correlation of sources in these shifts are non-zero and as different from each other as possible. The AMUSE algorithm consists of several steps. In the first step, data are gathered and a covariance matrix is estimated. Then follows a decomposition of singular values of the covariance matrix, estimation of number of sources, and dispersion of noise. Data are then transformed. The next step is eigenvalue/eigenvector decomposition. The source signal is estimated, followed by estimating the actual channel parameter matrix. More information and mathematical description could be found in [28].
- *ERICA algorithm* separates signals with non-zero kurtosis from mixed signals in the present of Gaussian noise. This algorithm is a quasi-Newton iteration, which will converge to saddle point with local isotropic convergence, regardless of source distribution. Whitening is not necessary for the algorithm to converge. The algorithm uses an iteration algorithm using a mixing matrix estimate, learning rate, and a matrix of fourth-order cross-cumulants for identifying the unknown of the mixing matrix. Demixing matrix is then computed as a pseudo-inverted matrix **A**. The iteration process continues until the selected convergence rate is achieved. More information and mathematical description can be found in [29].
- *FastICA algorithm* is currently the most commonly used type of the ICA method. It is built on a fixed iteration plan for finding the maximum data values that do not originate from normal data distribution. This can be achieved by using the approximation of Newton iteration. Pre-processing by centering and whitening must be done before applying the FastICA algorithm. Convergence criterion and maximum number of iterations must be set. The goal of the convergence is to achieve practically zero scalar product between the old and new vector values. First, random normed initial weights of the vector are created. A new vector is then calculated using the kurtosis and negentropy. This is followed by norming and checking whether the scalar product of the new vector and the initial weights vector is lower than the selected convergence criterion. If that is not the case, the second and third step of the FastICA method is repeated until the convergence criterion condition is satisfied or the selected number of iterations is exceeded [27].
- *FlexICA algorithm* is a learning algorithm with flexible linearity. This algorithm relies on hypothesized density functions since the probability density functions of source signals are unknown. It uses generalized Gaussian density, allowing approximation of all unimodal distributions. Simply speaking, FlexICA uses a combination of kurtosis and Gaussian exponentials to select the correct value of Gaussian exponentials. It can be defined as follows. As the first step, dimension is reduced by whitening. Then follows a search for orthogonality factor for minimization of common information in the whitened vector. This factor is then used to estimate the sought matrix **W**. More information and mathematical description can be found in [30].
- *Infomax algorithm* is based on maximizing the entropy and represents a natural gradient form for computing independent components. It can be considered as a neural network learning method. Infomax algorithm uses annealing based on weight changes to automate the separation process. The algorithm basically maps input values to output values so that the mean information between the input and output values is maximized. Learning rate function and function related to the nature of distribution are selected during the computation (i.e. super-Gaussian or sub-Gaussian). The output is a demixing matrix that is inverted mixing matrix. More information and mathematical description can be found in [31].
- *JADE algorithm* is based on common diagonalization of cumulation matrices. This algorithm is highly effective for separating low number of sources. Output components are separated by exploiting fourth-order moments, where orthogonal rotation of input signal is searched for the purpose of estimating source signals with high kurtosis. Firstly, the JADE algorithm estimates a whitening matrix. This is followed by estimating a maximum set of cumulation matrices. Orthogonal contrast is optimized by finding a rotation matrix so that the cumulation matrices are as diagonal as possible. Finally, the mixing matrix or direct output components (source signals) are estimated. More information and mathematical description can be found in [22].
- *KICA algorithm* is based on maximizing the kurtosis or, to be more precise, on optimization of kurtosis-based cost function. This cost function is identical to the function applied in the FastICA algorithm. The first step is centering and whitening of data. This is followed by initialization of a separation matrix and setting the required parameters. A rotation matrix is then created, followed by a computation of estimate component output and update of the demixing matrix. The components are estimated and the demixing matrix is updated by iterations. More information and mathematical description can be found in [32].
- *RADICAL algorithm* solves the ICA problem in arbitrary dimension. The RADICAL algorithm extracts independent signal sources using a differential entropy estimate based on spacing estimator. It is a consistent, asymptotically efficient, and computationally undemanding algorithm. The goal of the RADICAL algorithm is to minimize the contrast function using the Jacobean matrix, incorporated into the rotation matrix. The resulting rotation matrix contains all Jacobi rotations for all rotated pairs. This process is done for all sources estimated using the demixing matrix. The data must be whitened before applying the RADICAL. The advantage is its direct optimization of the statistic independence rate, absence of estimate of probability density and that it carries out one-dimensional entropy estimation, which converges quickly and avoids remote values. More information and mathematical description can be found in [33].
- *Robust ICA algorithm* is based on exact line search of optimal kurtosis. In this algorithm, the step size is optimized for each iteration in order to achieve kurtosis maximization and reduction of computational complexity. It is a simple modification of FastICA algorithm, where exact line search is in contract of the kurtosis. Several steps are taken during each iteration of optimal step-size optimization. Optimal step-size polynomial coefficients are computed in the first step, followed by extraction of step-size polynomial roots. In the next step, root leading to the absolute maximum is selected. The demixing matrix is then updated and normalization is performed. More information and mathematical description can be found in [34].
- *SIMBEC algorithm* uses simultaneous extraction of independent components that are present in the given data. The algorithm optimizes the maximum credibility criterion using a gradient algorithm on a Stiefel model. It uses natural gradient rise of Stiefel model to concurrently extract sources using a contrast function based on higher-order cumulants with a rate of learning that provides quick convergence. The algorithm is described by the following procedure. First, a separation matrix is computed. This is followed by a computation of cross-cumulant matrix. Finally, the searched source signals are estimated. More information and mathematical description can be found in [35].
- *SOBI algorithm* is based on second-order statistics for use of time-correlation structure to estimate of the original signals. The primary concept of SOBI is an assumption of diagonal form of delayed correlation matrices. This allows approximating the original signals. The input signals are whitened first, followed by sample covariance using input data and diagonalization. Noise power is then estimated by averaging the smallest eigenvalues in whitening matrix. The next step is to estimate unitary matrix in the joint diagonalization. Finally, the resulting matrix **A** is computed and source signals are estimated. More information and mathematical description can be found in [36].

### Fast transversal filter

The FTF is an adaptive filter that automatically modified filter coefficients so that the filter converges to optimal condition. This condition is achieved by minimizing the error signal between the adaptive filter output and the required signal [37]. In this case, mECG and aECG* signals estimated by the ICA method were used as inputs. The aECG* signal was considered as the desired signal $\vec{d}\left(n\right)$ and mECG signal was modified by adaptive filter into the form of maternal component in the aECG* signal. Such FTF-modified mECG signal was designated as $\vec{y}\left(n\right)$. By subtracting the signal $\vec{y}\left(n\right)$ from $\vec{d}\left(n\right)$, fECG considered as the error signal $\vec{e}\left(n\right)$ was found:

$$\begin{array}{c} \vec{e}\left(n\right)=\vec{d}\left(n\right)-\vec{y}\left(n\right). \end{array}$$ (4)

In case of FTF algorithm (as with recursive least squares algorithm), the error function is optimized by a deterministic approach. The main benefit of the FTF filter is its comparable performance with the recursive least squares method, while having shorter computation times (especially in higher orders of the filter). This is achieved by implementing four filters (forward prediction transversal filter, the backward prediction transversal filter, the gain computation transversal filter and the joint-process estimation transversal filter) operating together on a single task. However, this algorithm is burdened with instability in a finite precision environment. Description of this algorithm is rather comprehensive; see [37] for more detailed information.

### Dataset

All records of publicly available datasets (Labour and Pregnancy) were used for the experiments. The study protocol was approved by the Ethical Committee of the Silesian Medical University, Katowice, Poland (NN-013–345/02). Subjects read the approved consent form and gave written informed consent to participate in the study. We decided not to include other datasets because there are currently no sufficiently high-quality datasets on which it would be possible to objectively evaluate the quality of fECG extraction. Another reason is the need for expert-verified reference annotations so that the data can be properly validated. The selection of datasets was conditioned by the following criteria:

- *Dataset including real data*—the use of synthetic data for the experiments is not suitable because the algorithms often tend to work effectively, but when it is applied to real data, the algorithm often fails. Synthetically generated signals are therefore only suitable for initial experiments, not for the validation phase.
- *Availability of the fQRS annotations*—for an objective evaluation of the effectiveness of the algorithm, it is necessary to evaluate the correctness of determining the positions of fQRS complexes and estimating fHR. Without reference positions verified by experts, it is not possible to carry out objective assessment (using state-of-the-art evaluation metrics), and the effectiveness of the filtration can then only be evaluated subjectively, which is not very accurate.
- *Sufficient length and diversity of the recordings*—it is also important to use sufficiently long recordings (at least five minutes long) for testing. Testing only a short section of the signal can lead to distorted results. It is also important to include recordings of fetuses of different gestational ages, different ratios of mQRS:fQRS amplitudes and with different types and levels of interference. In addition to physiological records, it would be appropriate to use pathological records as well.

Although the availability of just such datasets is generally the biggest obstacle when testing algorithms for fECG extraction, the Labour and Pregnancy datasets come closest to the aforementioned requirements. Other publicly available datasets either contain synthetic signals, do not contain the reference positions of fQRS complexes, or contain very short and low-quality signals. All signals from Labour and Pregnancy dataset were acquired under clinical condition as part of research studies at the Department of Obstetrics and Gynaecology of the Medical University of Silesia in Katowice, Poland. The research was approved by the competent University Bioethics Committee and by participating patients. Records of both datasets are publicly available at *figshare repository*. All dataset information were acquired from [38].

In both datasets aECG signals have been sensed from the mother’s abdomen by means of four conventional Ag/AgCl electrodes and recorded using he KOMPOREL system consisting of signal recorder module and a portable computer. A conductive paste was applied on the upper epidermis layer before attaching the electrodes. The electrodes were attached around the mother’s navel line, common reference was placed above the *pubic symphysis*, and the electrode with active-ground signal was placed on the mother’s left leg. Direct fECG signal was sensed from the fetus’ head with a sterile spiral electrode. The signals were converted to a digital format with 16-bit resolution and sampling frequency of 500 Hz for aECG signals and of 1000 Hz for direct fECG. All sensed aECG signals have been pre-processed using a simple filter with multiple notches, located every 50 Hz. Low-frequency interference has been eliminated by setting the first frequency threshold to approx. 5 Hz; top-band between 45 Hz and 55 Hz was set to remove power-line interference. In direct fECG, the noise was suppressed in a comparable manner, but with modified filter parameters according to the sampling frequency [38].

- *Labour dataset*—the first used dataset contains twelve 5-minute long records of women between their 38th and 42nd week of pregnancy sensed in an advanced stage of labor. Each recording contains four aECG signals and includes a direct fECG signal sensed concurrently from the fetus’ head using a scalp electrode. Direct fECG gives the most reliable information about fHR and was therefore considered as reference or ‘gold standard’. In addition, the dataset includes annotation with precise positions of fQRS complexes determined by the authors to automatically detect R-peaks in direct fECG signal, correctness of which has been validated by clinical experts.
- *Pregnancy dataset*—the second used dataset contains ten 20-minute long records of women between their 32nd and 42nd week of pregnancy. Each recording also contains four aECG signals; in this case however, no reference direct fECG was sensed as the measurement using a scalp electrode can be done after the rupture of amniotic membrane. Nevertheless, in order to be able to use the signals for testing and evaluation the extraction algorithms, the authors processed and analyzed aECG signals and provided annotations with reference positions of mQRS and fQRS complexes. The correctness of fQRS complex position was again manually checked and corrected by clinical experts. Each fQRS complex was assigned with a reliability flag. Flag 1 means that the fQRS complex position was verified by a clinical expert, while flag 0 means that the position of the fQRS complex could not be verified by a clinical expert due to high levels of signal interference (number of unverified fQRS complexes was 130, i.e. 0.46% of the total number of marked fQRS complexes). The authors recommend excluding these unreliable fQRS complex positions when evaluating the detection accuracy.

The datasets used contain records acquired both during pregnancy and during childbirth. Therefore, they contain diverse signals varying in their quality, magnitudes of maternal and fetal components, as well as the interferences present. In the Labour dataset, the signals were recorded with a more prominent fetal component, while in the Pregnancy dataset, the fetal component was significantly lower compared to the maternal one. For example, for the Labour dataset, the mQRS:fQRS ratio was on average equal to 2 whereas for the Pregnancy dataset, the average mQRS:fQRS ratio was 3.5. Different levels and types of noise were present in both datasets. These were mainly power line interference, but also low-frequency interferences caused by movements (both maternal and fetal) or uterine contractions, as well as by impedance changes between the electrodes and maternal skin. Since the quality of individual records varied and cannot be generalized for the entire dataset, we present four parameters characterizing its quality for each record:

- *Parametr WM*—determining the mECG signal level in relation to interferences.
- *Parametr WF*—determining the fECG signal level in relation to interferences.
- *Parametr WEM*—characterizing energy changes of mQRS complexes in aECG signals.
- *Parametr WEF*—characterizing energy changes of fQRS complexes in aECG signals.

The WM and WF parameters assess the relationships of the amplitudes of the mECG and fECG signals in relation to the interferences in dB units. As for the WEM and WEF parameters, if there are no changes in the amplitude or shape of the individual mQRS/fQRS complexes, the values of the WEM and WEF parameters are equal to zero. The values of the WEM and WEF indices are also affected by the interference level. If the interference amplitude is comparable to the mQRS/fQRS amplitude, the WEF and WEF values take it into account. Detailed information about these parameters, including mathematical formulas for their calculation, can be found in the original publication by Matonia et al. [38]. The corresponding values of all selected parameters are shown for the Labour and Pregnancy dataset in Table 1. All available records (12 records from the Labour dataset and 10 records from the Pregnancy dataset) were used for our experiments and none of them were excluded.

**Table 1:** Values of parameters characterizing the quality of individual records from the labour and pregnancy dataset.

Record	Labour dataset				Pregnancy dataset			
	WM (dB)	WF (dB)	WEM (-)	WEF (-)	WM (dB)	WF (dB)	WEM (-)	WEF (-)
r1	10.6	6.7	0.20	0.20	16.5	5.3	0.05	0.45
r2	12.6	5.0	0.10	0.25	14.1	4.1	0.08	0.48
r3	7.8	-2.7	0.30	0.54	15.0	5.0	0.09	0.40
r4	11.7	2.3	0.13	0.31	12.5	4.0	0.14	0.51
r5	9.7	5.9	0.28	0.20	17.5	8.1	0.06	0.18
r6	12.8	3.7	0.10	0.19	12.9	4.4	0.11	0.31
r7	11.4	0.5	0.27	0.67	11.6	0.8	0.11	1.06
r8	10.6	7.0	0.18	0.18	10.9	-1.6	0.10	0.41
r9	11.4	3.9	0.15	0.44	16.9	-0.7	0.05	0.79
r10	11.6	0.7	0.11	0.48	14.9	5.1	0.07	0.59
r11	8.4	6.6	0.21	0.16	–	–	–	–
r12	6.5	3.8	0.36	0.28	–	–	–	–
Mean	10.4	3.6	0.20	0.33	14.3	3.4	0.09	0.52

### Evaluation parameters

It is highly desirable to maintain the same process of objective evaluation of extracted signals in order to be able to compare individual studies without having to repeat a certain experiment using already tested methods. Therefore, objective evaluation in this study is based on computation of R-peaks detection accuracy (ACC). This parameter is very often determined in various publications addressing fECG signal extraction and determination of R-peak positions, for example [18, 20]. Our team of authors focuses primarily on evaluations made with this parameter in large number of already published papers [15, 16].

In order to be able to compute the ACC parameter, the fECG signal must be first extracted and the position of R-peaks in it must be estimated. Additionally, the tested dataset must contain reference annotation of correct positions of R-peaks determined by the experts. The next step is to determine the true positive (TP), false positive (FP) and false negative (FN) parameters. Detected R-peaks in the extracted signal, which ranges within the interval of ±50 ms from the reference annotations, are marked as TP. Detected R-peaks in the extracted signal, which fall outside the mentioned interval, are marked as FP. And finally, any emitted R-peaks that should have been detected in the said interval, but were missing, are marked as FN. After the TP, FP, and FN parameters are determined, Eq (5) can be used to determine ACC, Eq (6) can be used to determine SE, Eq (7) to determine PPV, and finally Eq (8) to determine F1 [39].

$$\begin{array}{c} ACC=\frac{TP}{TP+FP+FN}\cdot 100\left(\%\right). \end{array}$$ (5)

$$\begin{array}{c} SE=\frac{TP}{TP+FN}\cdot 100\left(\%\right). \end{array}$$ (6)

$$\begin{array}{c} PPV=\frac{TP}{TP+FP}\cdot 100\left(\%\right). \end{array}$$ (7)

$$\begin{array}{c} F1=2\cdot \frac{SE\cdot PPV}{SE+PPV}\left(\%\right). \end{array}$$ (8)

### Experimental setup

**Fig 2:**
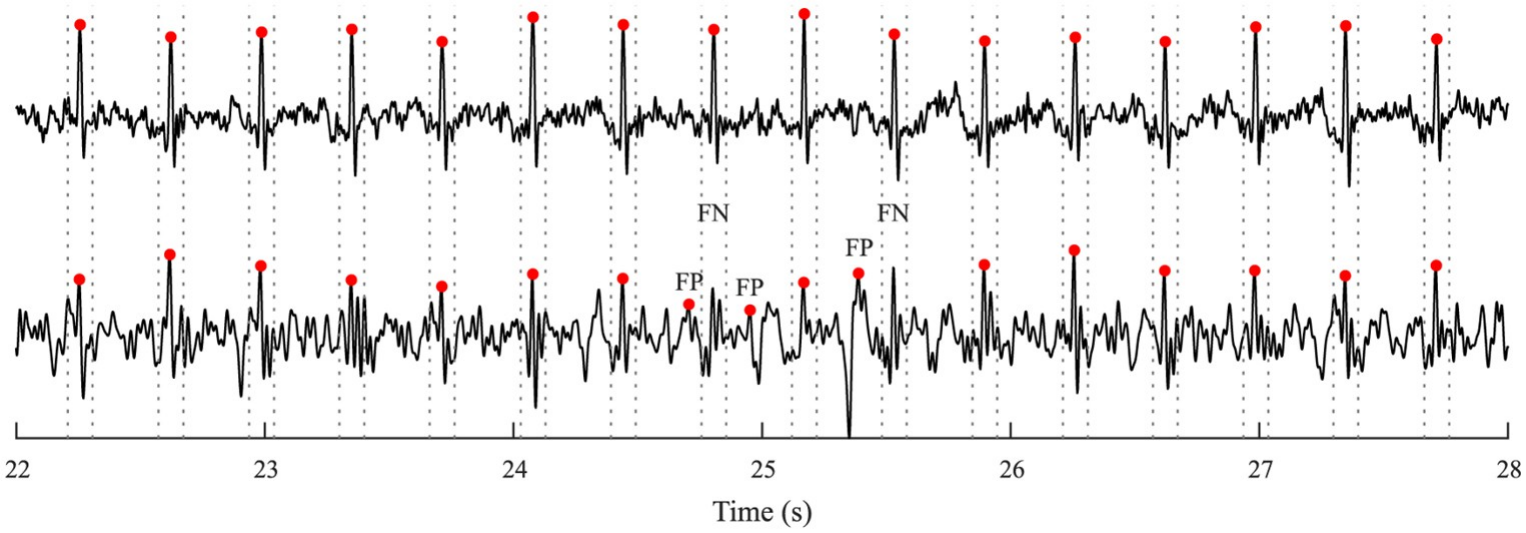
Comparison of the measured fECG signal using transvaginal scalp electrode with R-peaks highlighted by the annotations (upper curve) and the signal extracted with RADICAL-FTF with highlighted R-peaks detected by CWT detector (lower curve) on recording r6 of Labour dataset (grey dashed lines represent the intervals for determining TP).

Since ICA-based methods are multi-channel, at least two aECG signals were needed on the input side. It was found out based on our previous research [15, 16] that some aECG signals are not sensed with sufficient quality, which may lead to a needless deterioration of algorithm performance. For that reason, only 2 or 3 aECG input signals were used for further processing in some recordings (detailed description of the procedure for selecting optimal combinations of aECG signals for each recording is available for example in [15, 16]). For clarity reasons, the whole signal processing procedure is summarized in the following steps:

1. Pre-processing of aECG signals using a FIR filter to eliminate isoline fluctuations. Threshold frequencies were set to 5 through 50 Hz, the filter order was 500. The lower cutoff frequency of 5 Hz is chosen because the authors of the dataset removed frequencies below 5 Hz. The upper cutoff frequency was chosen at 50 Hz because the focus in this work is on fetal QRS complexes, which lie predominantly at frequencies 10–15 Hz [1].
2. Estimate of individual components (noise, mECG and aECG* signals with enhanced fetal component) from a mix of aECG signals using ICA-based methods. The number of output components was set to match the number of aECG input signals for the specific recording.
3. Automatic choice of aECG* and mECG together with amplitude/time centering. After receiving the output components from the ICA method, QRS complexes are detected in all output components. Based on the amplitudes of the QRS complexes, the polarity of the signals is adjusted. Subsequently, the one with the smallest number of QRS complexes and meeting the condition of 60—100 bpm is selected as the mECG signal. The remaining two output components are compared with each other using the SNR, where the signal with the higher SNR is designated as aECG*. Time centering is then performed, i.e. synchronization of the mECG and aECG* based on the positions of the detected QRS complexes. Finally, based on the amplitudes of the detected QRS complexes, the signals are equalized in amplitude. Both centerings are applied to increase the efficiency of the FTF algorithm.
4. Application of FTF algorithm used to modify the mECG signal in order for it to correspond to the mECG component in the aECG* signal as much as possible. The modified mECG signal was subtracted from aECG*, leading to fECG extraction.
5. Detection of R-peak in the resulting fECG signal using CWT detector [40]. The last step of the detector includes algorithm to modify the detected R-peaks based on 3 rules in accordance with patent published in [41]:
  - Missing R-peak is added if the current RR interval > 1.3 times the median of all RR intervals. In such case, the missing R-peak is added right in the middle between adjacent R-peaks.
  - Incorrectly detected R-peak is removed if the current RR interval < 0.7 times the median of all RR intervals. In such case, the excess R-peak is removed.
  - R-peak is determined as incorrectly positioned if the RR_i_ interval < 0.9 times the median of RR intervals and concurrently RR_i_+1 interval > 1.1 times the median of RR intervals. This goes vice versa—the RR_i_ interval > 1.1 times the median of RR intervals and concurrently RR_i_+1 interval < 0.9 times the median of RR intervals. In such case, the incorrect position of QRS complex shifted right in the middle between adjacent R-peaks.
  The example of detected R-peak in recording r6 of the Labour dataset compared to direct fECG with highlighted reference positions of R-peaks by annotations is shown in Fig 2. The direct fECG signal could not be acquired in Pregnancy dataset. In example, the grey dashed lines represent the ±50 ms interval from reference annotations for marking the detected R-peak as a TP value.
6. Determination of TP, FP, and FN with estimated R-peak position and reference R-peak positions in annotations. Computation of ACC, SE, PPV, and F1 parameters based on the determined TP, FP, and FN values.

## Results

This section summarizes the results of fQRS complex detection achieved by each ICA-based method and subsequent application of an adaptive FTF filter. The ICA-based methods were also compared in terms of time complexity. In this section, a statistical analysis is presented to determine whether the differences between the tested algorithms are statistically significant, both in terms of the quality of filtration they are providing, and in terms of their time consumption. All data used and analysed are available as supporting material to this study.

### Accuracy of fQRS complex detection

The resulting ACC values acquired with each ICA-based method and the follow-up application of FTF are summarized for Labour dataset in Table 2. According to our subjective assessment, the performance of individual algorithms was very similar for some records but differed for the others. For the Labour dataset, six records (r1, r5, r6, r8, r11 and r12) achieved very high accuracies using all tested methods, and the ACC value did not differ by more than 6%, which can be considered a negligible difference. These were high-quality captured aECG signals and extraction was not difficult for any algorithm. For the r2 recording, the results of all algorithms were similar, except for the RobustICA and ERICA algorithms, which had the largest percentage difference (ERICA achieved 11.86% lower transmission than RobustICA), which was due to ERICA’s inability to accurately detect fQRS complexes. On the other hand, in the case of record r3, the RobustICA method achieved the worst result (28.94%). Since this was a signal of lower quality, the method probably failed to recognize the fetal component and considered it as interference. The KICA method coped best with this problematic signal and can therefore be recommended for processing both high-quality and low-quality input signals. RobustICA, together with ERICA, also failed for recording r4, in which they also failed to correctly recognize the fetal component and labeled it as interference, and considered the mQRS residue or other interference as a fetal component and labeled it as fQRS. A difference of 29.06% between the most efficient method (RADICAL) and the worst method (SIMBEC) was observed for the r7 record, for which the SIMBEC method was unable to suppress residues of mQRS complexes. The same problem also occurred with the r9 record, where the JADE method failed compared to the RADICAL method, resulting in an accuracy difference of 11.98%. For the r10 record, the difference in accuracy between the best (RADICAL) and the worst (SIMBEC) method was even higher, at 22.25%.

**Table 2:** Statistical evaluation of fQRS complex detection based on the ACC (%) parameter for each ICA-based method and the follow-up FTF applications in recordings of the labour dataset.

Record	AMUSE	ERICA	FastICA	FlexICA	Infomax	JADE	KICA	RADICAL	RobustICA	SIMBEC	SOBI
r1	95.60	98.92	98.92	98.92	**99.23**	98.92	98.92	98.92	98.92	98.92	98.92
r2	89.90	81.72	91.30	92.15	86.37	92.43	93.42	91.58	**93.58**	90.88	91.58
r3	40.72	44.64	46.51	46.20	48.10	47.50	**48.15**	47.91	28.94	47.39	46.38
r4	79.11	48.75	**88.83**	88.31	87.55	77.03	85.64	75.58	48.10	84.66	87.55
r5	92.54	96.13	96.57	**97.75**	96.57	97.01	97.01	97.01	97.01	97.16	95.55
r6	97.69	96.28	96.56	97.83	93.23	96.98	97.55	97.55	**97.98**	95.30	97.55
r7	87.00	80.34	82.42	86.14	85.51	81.99	82.16	**95.07**	81.66	69.01	85.86
r8	**100.00**	**100.00**	**100.00**	**100.00**	**100.00**	**100.00**	**100.00**	**100.00**	**100.00**	**100.00**	**100.00**
r9	91.36	87.67	89.50	95.80	89.11	84.53	93.56	**96.51**	92.45	85.36	95.93
r10	97.64	87.09	86.92	94.12	91.03	85.27	86.26	**98.89**	83.45	76.64	94.42
r11	97.87	97.87	97.56	97.56	97.87	97.87	97.56	97.87	97.87	97.87	**98.02**
r12	98.34	**100.00**	**100.00**	**100.00**	**100.00**	**100.00**	**100.00**	**100.00**	**100.00**	**100.00**	98.79
Mean	88.98	84.95	89.59	91.23	89.55	88.29	90.02	**91.41**	85.00	86.93	90.88

As for the average values, in all cases exceeded 80% (i.e. mean ACC values of all methods ranged between 84.95% and 91.41%), and thus all tested algorithms achieved effective extraction in this dataset. When assessing the effectivity of the algorithms based on the mean of the ACC values over all recordings, the most effective algorithm is the RADICAL (91.41%), followed by FlexICA, SOBI and KICA methods that reached mean accuracy of over 90% (91.23%, 90.88%, and 90.02%, respectively). As the least effective methods, we can consider the methods with the mean accuracy below 90%, i.e. ERICA, Robust ICA, and SIMBEC, reaching 84.95%, 85%, and 86.93%, respectively.

For a detailed statistic results (including TP, FP, FN values and ACC, SE, PPV, F1 indices) achieved by the most effective RADICAL method for each recording, see Table 3. The table includes a combination of the used aECG input signals. It shows that the method was effective from the perspective of ACC (ACC > 80%) in most recordings, except r3 and r4. SE, PPV, and F1 parameters exceeded 80% in all recordings except r3. In additions, all fQRS complexes were correctly detected in recordings r10 and r12 (no FP or FN values were detected) and all parameters reached 100.00%.

**Table 3:** Statistical evaluation of fQRS complex detection based on the ACC, SE, PPV, and F1 parameters for the RADICAL algorithm and the follow-up FTF application in recordings of the labour dataset.

Record	Combination of electrodes	Number of R-peaks by annotations	TP	FP	FN	ACC (%)	SE (%)	PPV (%)	F1 (%)
r1	1, 3, 4	644	641	4	3	98.92	99.53	99.38	99.46
r2	1, 3, 4	660	620	40	17	91.58	97.33	93.94	95.61
r3	1, 3, 4	684	505	338	211	47.91	70.53	59.91	64.79
r4	1, 2, 4	632	591	101	90	75.58	86.78	85.40	86.09
r5	1, 3, 4	645	649	9	11	97.01	98.33	98.63	98.48
r6	1, 3, 4	674	676	9	8	97.55	98.83	98.69	98.76
r7	2, 3, 4	627	617	17	15	95.07	97.63	97.32	97.47
r8	2, 3, 4	651	645	0	0	100.00	100.00	100.00	100.00
r9	1, 2, 4	657	663	13	11	96.51	98.37	98.08	98.22
r10	2, 3, 4	637	624	4	3	98.89	99.52	99.36	99.44
r11	1, 2, 3	705	642	10	4	97.87	99.38	98.47	98.92
r12	1, 2, 4	685	657	0	0	100.00	100.00	100.00	100.00

The ACC values acquired for the Pregnancy dataset with each ICA-based method and the follow-up application of FTF are summarized in Table 4. For this dataset, the algorithms achieved high quality results only for four records (r1, r4, r5, and r10), while their ACC values did not differ by more than 8.13%. For the r2 record, the results of the individual algorithms were comparable, but a significant difference in accuracy (26.48%) was achieved with the RADICAL and RobustICA methods. The reason was that the RobustICA was not able to correctly recognize the fetal component and labeled the interference or the maternal component as fQRS complexes. The same case also occurred with records r3, r7, and r9, where one method deviated. For the r3 record, it was the SIMBEC method with a difference of 21.62% compared to the most effective KICA. For the r7 record, the least effective AMUSE algorithm differed from the most effective one (ERICA) by 21.23%. As for the r9 record, it was ERICA achieving lower accuracy by up to 36.42% compared to KICA, the most effective algorithm for this record. On the other hand, for records r6 and r8, almost all methods achieved very inaccurate extraction (for r6 the ACC values were in the range 14.47%–59.08% and for r8 in the range 24.17%–66.56%), which was due to the low quality of input aECG signals. In the case of the r6 recording, the RobustICA method was able to best distinguish noise from the useful fECG signal while in the case of the r8 recording, it was the FastICA method.

**Table 4:** Statistical evaluation of fQRS complex detection based on the ACC (%) parameter for each ICA-based method and the follow-up FTF applications in recordings of the pregnancy dataset.

Record	AMUSE	ERICA	FastICA	FlexICA	Infomax	JADE	KICA	RADICAL	RobustICA	SIMBEC	SOBI
r1	89.09	96.91	96.91	96.72	96.91	96.91	96.94	**97.22**	96.91	96.60	96.60
r2	85.25	88.13	80.62	85.32	89.99	87.26	77.22	**93.30**	66.82	86.61	80.50
r3	88.77	90.94	93.82	94.41	93.86	94.01	**95.34**	89.79	86.14	73.72	94.26
r4	**98.14**	96.01	97.90	**98.14**	**98.14**	96.64	94.16	97.83	93.85	98.00	97.90
r5	99.53	99.75	99.71	99.78	99.75	99.71	99.75	99.78	99.75	**99.82**	99.53
r6	32.40	57.41	53.26	15.30	28.73	55.17	56.89	14.47	**59.08**	44.92	15.73
r7	60.55	**81.78**	79.16	81.00	80.01	80.87	80.48	79.08	80.98	76.04	78.02
r8	45.20	54.13	**66.56**	44.68	24.17	42.29	41.62	44.56	42.22	33.49	32.58
r9	91.96	60.91	91.99	95.47	96.58	76.37	**97.33**	92.45	95.23	96.01	96.24
r10	82.51	80.51	81.45	81.02	81.88	76.70	81.76	78.58	**84.40**	83.15	82.17
Mean	77.34	80.65	**84.14**	79.18	79.00	80.59	82.15	78.70	80.54	78.84	77.35

The extraction accuracy was generally lower in this dataset, as the mean ACC values ranged between 77.34%–84.14%, and 6 variants of the ICA method (AMUSE, FlexICA, Infomax, RADICAL, SIMBEC, SOBI) did not exceed the mean ACC of 80%. JADE and SOBI methods were not the most accurate in fQRS complex detection in none of the recordings and had mean values of ACC = 80.59% and ACC = 77.35%, respectively. AMUSE, ERICA, FlexICA, Infomax, and SIMBEC algorithms achieved the most accurate detection of fQRS complexes with 1 recording with mean values of ACC = 77.34%, ACC = 80.65%, ACC = 79.18%, ACC = 79%, and ACC = 78.84%, respectively. The most effective methods were KICA, RADICAL, and RobustICA, which achieved the most accurate detection of fQRS complexes with 2 recordings with mean values of ACC = 82.15%, ACC = 78.70% and ACC = 80.54%, respectively. FastICA turned out to be the most suitable for Pregnancy dataset; even though it achieved the most accurate detection of fQRS complexes with 1 recording only, it had the highest mean value of ACC (ACC = 84.14%).

Even in this dataset, detailed statistical results with all recordings achieved with the most effective method, FastICA, are provided; see Table 5. From the perspective of the ACC parameter, the algorithm was effective (ACC > 80%) in most of the recordings, with the exception of r6, r7, and r8. The SE value exceeded 80% in all recordings except r6 and PPV, and F1 parameter values were higher than 80% with most recordings except r6 and r8. In this case, efforts to correctly detect all fQRS complexes failed in all recordings. In other words, a 100.00% accuracy was not achieved in none of the recordings.

**Table 5:** Statistical evaluation of fQRS complex detection based on the ACC, SE, PPV, and F1 parameters for the FastICA algorithm and the follow-up FTF application in recordings of the pregnancy dataset.

Record	Combination of electrodes	Number of R-peaks by annotations	TP	FP	FN	ACC (%)	SE (%)	PPV (%)	F1 (%)
r1	2, 4	3118	3073	53	45	96.91	98.56	98.30	98.43
r2	1, 4	2791	2534	352	257	80.62	90.79	87.80	89.27
r3	1, 2, 3, 4	2557	2473	79	84	93.82	96.71	96.90	96.81
r4	2, 3, 4	2774	2747	32	27	97.90	99.03	98.85	98.94
r5	3, 4	2764	2764	8	0	99.71	100.00	99.71	99.86
r6	1, 4	2879	2041	953	838	53.26	70.89	68.17	69.50
r7	1, 2	3096	2773	407	323	79.16	89.57	87.20	88.37
r8	1, 4	2897	2351	635	546	66.56	81.15	78.73	79.93
r9	3, 4	2816	2735	157	81	91.99	97.12	94.57	95.83
r10	1, 4	2583	2384	344	199	81.45	92.30	87.39	89.78

**Fig 3:**
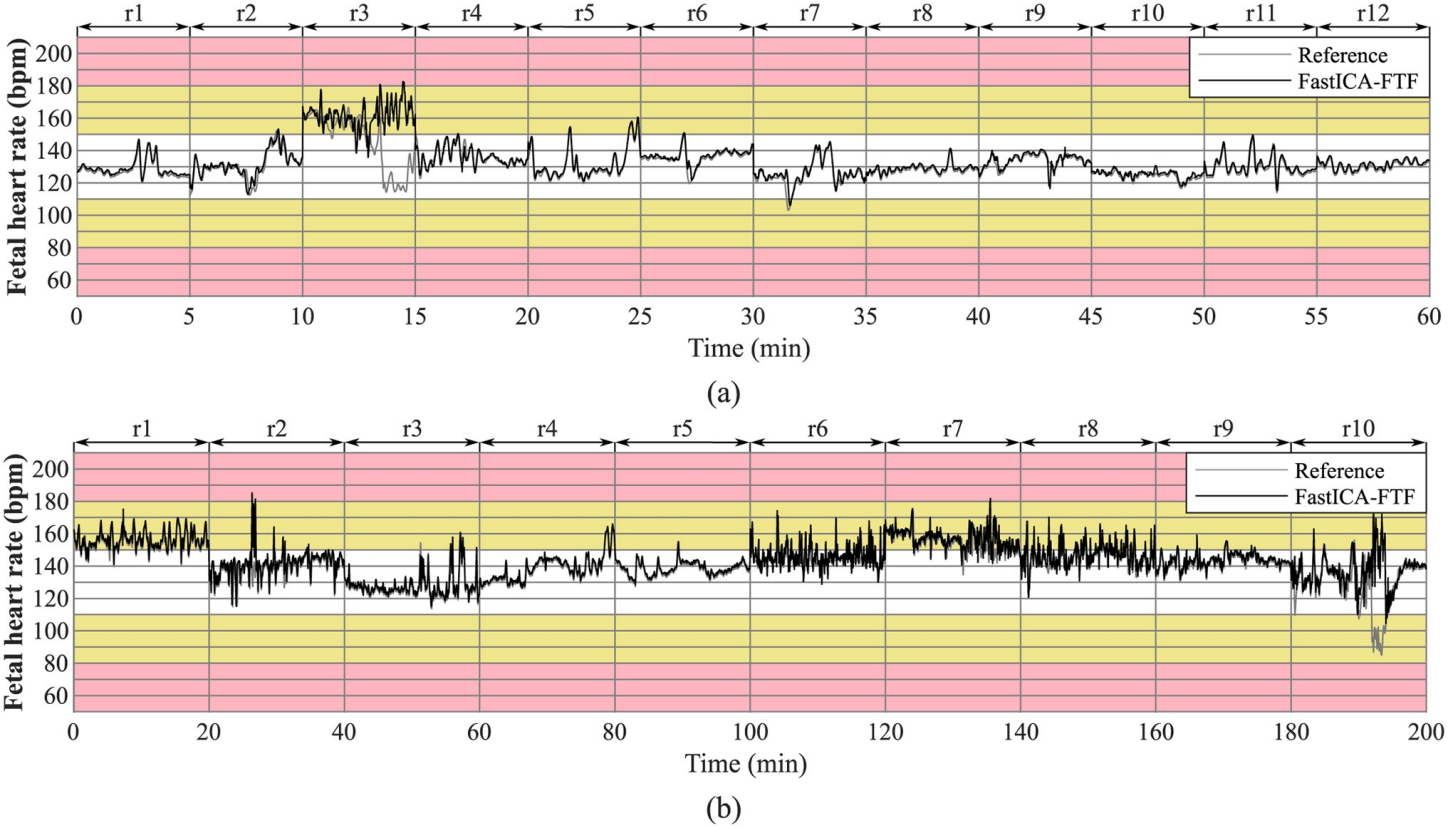
Comparison of fHR traces created from signals extracted with the FastICA-FTF method and reference fHR trace for all recordings. (a) Labour dataset and (b) Pregnancy dataset.

The main reason for the detection of the fQRS complexes is their use to determine fHR, which is then used by the clinicians to determine fetal health state. Therefore, we provide an example of a fHR trace created from signals extracted with the FastICA-FTF compared to the reference fHR trace for all recordings of the Labour dataset (Fig 3a) and Pregnancy dataset (Fig 3b) is shown in Fig 3. A moving average with a window length of 15 was applied to the reference and extracted fHR signal. This is because usually the fHR of the extracted signal is not so accurate and tends to produce positive or negative peaks. In the case of Labour dataset, fHR traces were estimated with FastICA-FTF method in all recordings, except recording r3 comparable to the reference fHR traces. In case of Pregnancy dataset, the trend of reference fHR traces was followed in all recordings, with the exception of recordings r6, r7, r8, and r10, the estimated fHR traces slightly differed from the references. Less accurate determination of the fHR traces in records of both datasets were caused by low-quality aECG signals. It would also be beneficial if measuring system would use abdominal electrodes only, i.e. without the need to sense the reference chest signals, which would be comfortable and stress-reducing for the mother.

### Evaluation of algorithms time complexity

In addition to evaluation of algorithms from the perspective of extraction quality, we compared each ICA-based method from the perspective of computation time; see Table 6. In order for this experiment to be objective, all algorithms were run on the same PC with the following configuration: Core i9 9980XE (18c/36t), MEG X299 CREATION, 64GB DDR4 3200MHz cl14, GTX 1050 TI. Only one test of ICA-based method was running at one time and no other operations were running in the background that would reduce the performance (only basic applications were running in the background without any greater effect on the performance). Further, for each ICA-based method, we performed the tests on each record for all possible combinations of 2 inputs (6 combinations), then 3 inputs (4 combinations), and finally 4 inputs (1 combination). We then performed the median of all estimated times for 2 inputs, 3 inputs and 4 inputs. For each input count option, on average the experiment was run more than 40 times (depending on the number of records in the dataset and the combination). The effect of number of input signals to each ICA-based method was also observed and this experiment was made separately on both tested datasets so that the effect of the different number of input signal samples can be seen.

**Table 6:** Median duration of individual ICA-based methods for different recording lengths and different number of inputs.

Algorithm	Time (s)						
	150000 (Labour dataset)			598900 (Pregnancy dataset)			Mean
	2	3	4	2	3	4	
AMUSE	0.010	0.012	0.012	0.023	0.033	0.035	0.021
ERICA	0.726	1.081	0.887	1.423	2.469	5.889	2.079
FastICA	0.178	0.229	0.263	0.292	0.659	1.090	0.452
FlexICA	0.277	0.542	0.530	1.165	2.428	3.696	1.440
Infomax	7.529	7.469	7.624	32.771	32.822	44.091	22.051
JADE	0.014	0.026	0.039	0.031	0.071	0.106	0.048
KICA	0.012	0.014	0.015	0.020	0.028	0.031	**0.020**
RADICAL	1.435	7.933	19.154	4.486	24.447	64.829	20.381
RobustICA	0.250	0.360	0.338	0.148	0.871	0.750	0.453
Simbec	0.189	0.363	0.392	0.236	0.704	1.397	0.547
SOBI	0.427	0.514	0.518	1.341	1.525	1.792	1.020

According to the recorded computation times shown in Table 6, the fastest method was the KICA method with mean computation time of 0.02 s. The slowest were the Infomax and RADICAL method with the mean computation time exceeding 20 s. The FastICA method, which achieved the most effective extraction, was the sixth fastest with mean time of 0.452 s. However, the difference between the computation time of the FastICA method and the fastest AMUSE and KICA methods was merely several tenths of a second, which can be deemed insignificant. When comparing the computation times of the FastICA and the RADICAL method, which also achieved very good extraction results, the FastICA can be considered superior with its almost fifty times shorter computation time compared to the RADICAL. In addition, the computation time of the RADICAL method was substantially rising with the increasing number of aECG input signals. This might obstruct efforts to implement this method into a device operating in real-time, which often utilizes higher number (up to tens) of sensing electrodes.

### Statistical analysis

In order to find out whether the differences between the tested algorithms are statistically significant, we performed a) a statistical analysis of the results achieved for all used parameters evaluating the quality of filtration (ACC, SE, PPV and F1) and b) a statistical analysis of the time consumption of individual algorithms. Statistical analysis was performed using *R Core Team*. In all cases, statistical significance was set as p < 0.05.

First, the normality of the data was tested using the Shapiro-Wilk test for all parameters evaluating the quality of filtration, as well as for the time requirements for each algorithm. In some cases, statistically significant deviations from normality were detected, and therefore non-parametric methods were chosen for data description and subsequent analysis. The median and interquartile range (IQR) were used to describe the analyzed variables. A detailed analysis of the filtration quality results was performed for individual evaluation parameters and individual datasets. However, the results did not show significant differences between the given results and for this reason only the summary analysis for both datasets and one selected parameter (ACC) is presented. A deeper analysis, separately for the Labour dataset and separately for the Pregnancy dataset, is then devoted to the analysis of the time requirements of the tested algorithms, where statistically significant differences were found.

To statistically evaluate the differences between the tested algorithms in terms of their filtration outputs and the computational demands, we used the Friedman test, which is the non-parametric alternative to the one-way ANOVA with repeated measures, supplemented with the Kendall concordance coefficient. The Kendall concordance coefficient expresses the simultaneous association (relatedness) between k sets of rankings (i.e., cases; correlated samples). The range of Kendall concordance is from 0 to +1. Values close to zero represent lack of agreement in the rankings of the algorithms among records, while values close to 1 represent perfect agreement in the rankings of the algorithms among records. In case of the Friedman test detecting a statistically significant difference between individual algorithms, a Conover post-hoc analysis with Benjamini & Yekutieli correction was performed to calculate dusted p-values for the detection of homogeneous subgroups of algorithms.

**Fig 4:**
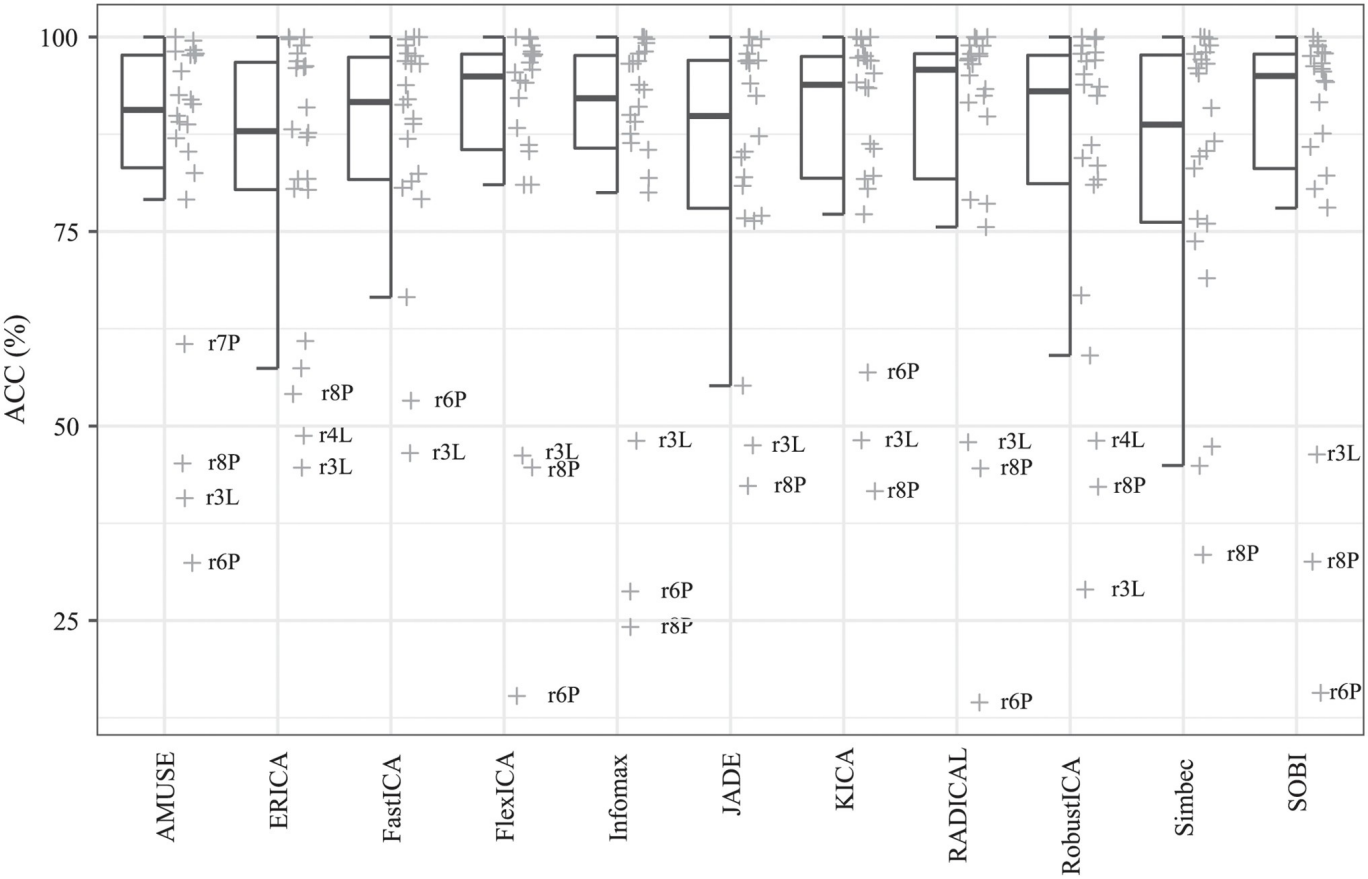
Hybrid boxplots providing comparison of the detection assessed by ACC parameter for all compared algorithms.

1. *Detection accuracy results*
  When comparing parameters evaluating the quality of filtering records (ACC, SE, PPV, F1) across both datasets, no statistically significant differences between the tested algorithms were found (in all cases p-value > 0.05), see Table 7. Therefore, we decided to further analyze the results of the comparison results only for one of these parameters, namely ACC, see Fig 4. It can be noted that for ACC there are outliers for all algorithms, while they all belong to the same records: for the Labour dataset they are records r3 and r4, and for the Pregnancy dataset they are records r6 and r8. These deviations are probably related to the reduced quality of the input signals.
2. *Time consumption analysis results*
  In the next step, we analyzed the time consumption of the tested algorithms. For each algorithm, the time needed to create ICA components was measured with different numbers of inputs (for example, 3 tested scenarios: 2 inputs, 3 inputs, 4 inputs). This methodology was selected because the number of inputs significantly affects the quality of the extraction and the computational complexity. The results showed a statistically significant difference between the calculation times for the individual algorithms for all tested scenarios (in all cases the p-value for both the Labour dataset and the Pregnancy dataset was < 0.001), see Tables 8 and 9, respectively.

**Table 7:** Statistical analysis of the results of all tested algorithms evaluated using ACC, SE, PPV and F1 parameters for both selected datasets.

Algorithm	ACC (%)	F1 (%)	PPV (%)	SE (%)
	Median (IQR)	Median (IQR)	Median (IQR)	Median (IQR)
AMUSE	90.63 (83.19; 97.67)	95.08 (90.82; 98.82)	94.38 (88.69; 98.59)	95.80 (93.01; 98.87)
ERICA	87.90 (80.38; 96.75)	93.56 (89.12; 98.35)	92.83 (87.24; 98.27)	94.31 (89.69; 98.48)
FastICA	91.65 (81.69; 97.40)	95.64 (89.92; 98.68)	94.34 (88.41; 98.33)	96.92 (91.17; 98.91)
FlexICA	94.94 (85.52; 97.81)	97.40 (92.19; 98.89)	97.06 (91.52; 98.91)	97.86 (92.57; 99.04)
Infomax	92.13 (85.72; 97.63)	95.90 (92.31; 98.80)	95.72 (91.19; 98.43)	96.13 (92.89; 99.21)
JADE	89.85 (77.99; 97.00)	94.63 (87.62; 98.48)	93.29 (86.89; 98.43)	95.63 (89.61; 98.65)
KICA	93.86 (81.86; 97.49)	96.83 (90.02; 98.73)	96.58 (88.58; 98.55)	97.34 (90.92; 99.13)
RADICAL	95.79 (81.75; 97.86)	97.85 (89.89; 98.92)	97.70 (88.88; 98.81)	97.98 (91.59; 99.27)
RobustICA	93.02 (81.15; 97.65)	96.38 (89.59; 98.81)	95.43 (88.66; 98.59)	96.58 (90.27; 99.00)
Simbec	88.74 (76.19; 97.69)	94.02 (86.49; 98.83)	92.56 (85.34; 98.59)	95.53 (87.49; 99.02)
SOBI	94.98 (83.09; 97.81)	97.43 (90.76; 98.89)	97.12 (88.97; 98.63)	97.45 (92.39; 99.05)
Friedman test (p-value)	0.253	0.253	0.148	0.230
Kendall tau	0.057	0.057	0.066	0.059

**Table 8:** Statistical analysis of time requirements for different number of inputs depending on the compared algorithms for labour dataset.

Algorithm	Time (s)		
	2 inputs	3 inputs	4 inputs
	Median (IQR)	Median (IQR)	Median (IQR)
AMUSE	0.010 (0.010; 0.010)	0.012 (0.012; 0.012)	0.012 (0.012; 0.012)
ERICA	0.602 (0.487; 0.649)	0.827 (0.635; 1.013)	0.745 (0.587; 0.914)
FastICA	0.110 (0.078; 0.156)	0.233 (0.186; 0.255)	0.205 (0.197; 0.300)
FlexICA	0.243 (0.193; 0.281)	0.463 (0.369; 0.687)	0.452 (0.395; 0.506)
Infomax	7.519 (7.450; 7.582)	7.470 (7.381; 7.557)	7.659 (7.565; 7.710)
JADE	0.014 (0.014; 0.014)	0.026 (0.025; 0.026)	0.039 (0.038; 0.039)
KICA	0.012 (0.012; 0.012)	0.014 (0.014; 0.014)	0.015 (0.015; 0.015)
RADICAL	1.437 (1.415; 1.454)	7.943 (7.850; 8.018)	19.171 (18.874; 19.366)
RobustICA	0.064 (0.062; 0.079)	0.176 (0.167; 0.203)	0.330 (0.281; 0.364)
Simbec	0.174 (0.141; 0.246)	0.363 (0.211; 0.467)	0.260 (0.213; 0.347)
SOBI	0.427 (0.426; 0.428)	0.514 (0.513; 0.514)	0.516 (0.515; 0.518)
Friedman test (p-value)	< 0.001^a^	< 0.001^b^	< 0.001^c^
Kendall tau	0.946	0.945	0.948

Friedman test, Conover Post Hoc analysis:

^*a*^- (AMUSE, KICA, JADE, RobustICA, FastICA) vs. (KICA, JADE, RobustICA, FastICA, Simbec) vs. (JADE, RobustICA, FastICA, Simbec, FlexICA, SOBI) vs. (RobustICA, FastICA, Simbec, FlexICA, SOBI, ERICA) vs. (FastICA, Simbec, FlexICA, SOBI, ERICA, RADICAL) vs. (FlexICA, SOBI, ERICA, RADICAL, Infomax),

^*b*^- (AMUSE, KICA, JADE, RobustICA, FastICA) vs. (KICA, JADE, RobustICA, FastICA, Simbec); (JADE, RobustICA, FastICA, Simbec, FlexICA, SOBI) vs. (RobustICA, FastICA, Simbec, FlexICA, SOBI, ERICA) vs. (Simbec, FlexICA, SOBI, ERICA, Infomax, RADICAL),

^*c*^- (AMUSE, KICA, JADE, FastICA, Simbec, RobustICA) vs. (KICA, JADE, FastICA, Simbec, RobustICA, FlexICA, SOBI) vs. (FastICA, Simbec, RobustICA, FlexICA, SOBI, ERICA) vs. (Simbec, RobustICA, FlexICA, SOBI, ERICA, Infomax) vs. (FlexICA, SOBI, ERICA, Infomax, RADICAL)

**Table 9:** Statistical analysis of time requirements for different number of inputs depending on the compared algorithms for pregnancy dataset.

Algorithm	Time (s)		
	2 inputs	3 inputs	4 inputs
	Median (IQR)	Median (IQR)	Median (IQR)
AMUSE	0.023 (0.023; 0.023)	0.032 (0.032; 0.032)	0.035 (0.034; 0.035)
ERICA	0.997 (0.984; 1.515)	2.069 (1.758; 2.729)	2.841 (1.539; 4.299)
FastICA	0.229 (0.202; 0.288)	0.635 (0.442; 0.874)	1.028 (0.535; 1.503)
FlexICA	1.071 (0.855; 1.304)	2.285 (1.744; 2.808)	3.076 (2.446; 4.964)
Infomax	32.797 (32.172; 33.286)	32.643 (32.326; 33.033)	43.399 (43.187; 44.477)
JADE	0.030 (0.030; 0.031)	0.071 (0.070; 0.071)	0.106 (0.105; 0.108)
KICA	0.020 (0.020; 0.020)	0.028 (0.028; 0.029)	0.031 (0.031; 0.031)
RADICAL	4.485 (4.414; 4.532)	24.461 (24.212; 24.735)	64.948 (63.820; 65.278)
RobustICA	0.145 (0.141; 0.158)	0.357 (0.341; 0.408)	0.670 (0.605; 0.832)
Simbec	0.252 (0.195; 0.278)	0.554 (0.426; 0.766)	1.099 (0.622; 1.432)
SOBI	1.341 (1.339; 1.343)	1.526 (1.525; 1.527)	1.793 (1.790; 1.794)
Friedman test (p-value)	< 0.001^a^	< 0.001^b^	< 0.001^c^
Kendall tau	0.975	0.950	0.940

Friedman test, Conover Post Hoc analysis:

^a^- (AMUSE, KICA, JADE, RobustICA, FastICA) vs. (KICA, JADE, RobustICA, FastICA, Simbec) vs. (JADE, RobustICA, FastICA, Simbec, FlexICA, SOBI) vs. (RobustICA, FastICA, Simbec, FlexICA, SOBI, ERICA) vs. (FastICA, Simbec, FlexICA, SOBI, ERICA, RADICAL) vs. (FlexICA, SOBI, ERICA, RADICAL, Infomax),

^b^- (AMUSE, KICA, JADE, RobustICA, FastICA) vs. (KICA, JADE, RobustICA, FastICA, Simbec); (JADE, RobustICA, FastICA, Simbec, FlexICA, SOBI) vs. (RobustICA, FastICA, Simbec, FlexICA, SOBI, ERICA) vs. (Simbec, FlexICA, SOBI, ERICA, Infomax, RADICAL),

^c^- (AMUSE, KICA, JADE, FastICA, Simbec, RobustICA) vs. (KICA, JADE, FastICA, Simbec, RobustICA, FlexICA, SOBI) vs. (FastICA, Simbec, RobustICA, FlexICA, SOBI, ERICA) vs. (Simbec, RobustICA, FlexICA, SOBI, ERICA, Infomax) vs. (FlexICA, SOBI, ERICA, Infomax, RADICAL)

**Fig 5:**
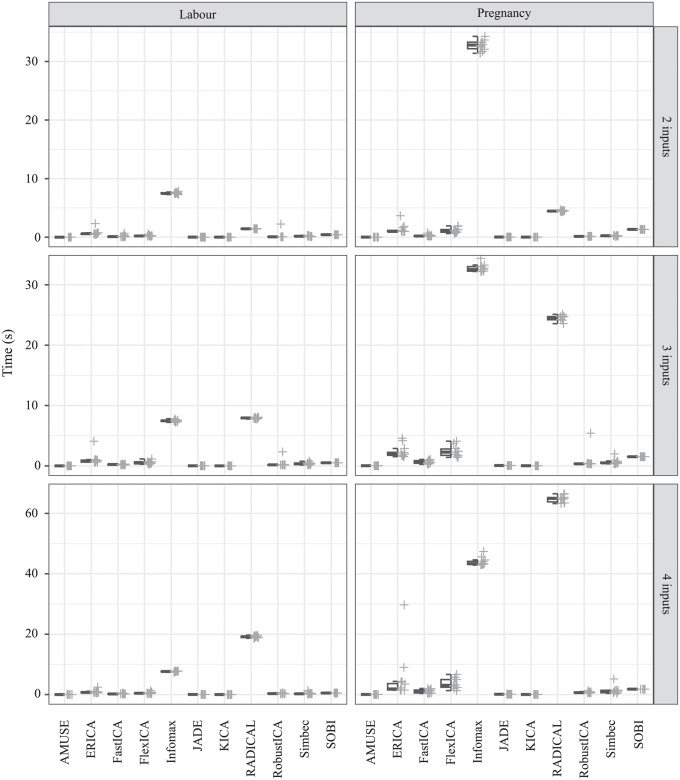
Hybrid boxplots providing comparison of time requirements for all tested algorithms separately for both datasets and for different number of inputs.

Fig 5 shows a comparison of the required times of individual algorithms using hybrid boxplots. The comparison was made separately for individual numbers of inputs and also separately for the Labour dataset and the Pregnancy dataset. In all cases, it can be seen that the Infomax and RADICAL algorithms show noticeably longer times, which was also confirmed by the results of the Conover post-hoc analysis, which always included these algorithms in the group of the slowest algorithms. In contrast, the AMUSE, KICA, and JADE algorithms appear in most groups associated with the shortest times. Furthermore, there is a noticeable influence of the input data, where the results show longer calculation times for records from the Pregnancy dataset. In the statistical analysis of parameters evaluating the quality of filtration, outliers from various records can be noted.

**Fig 6:**
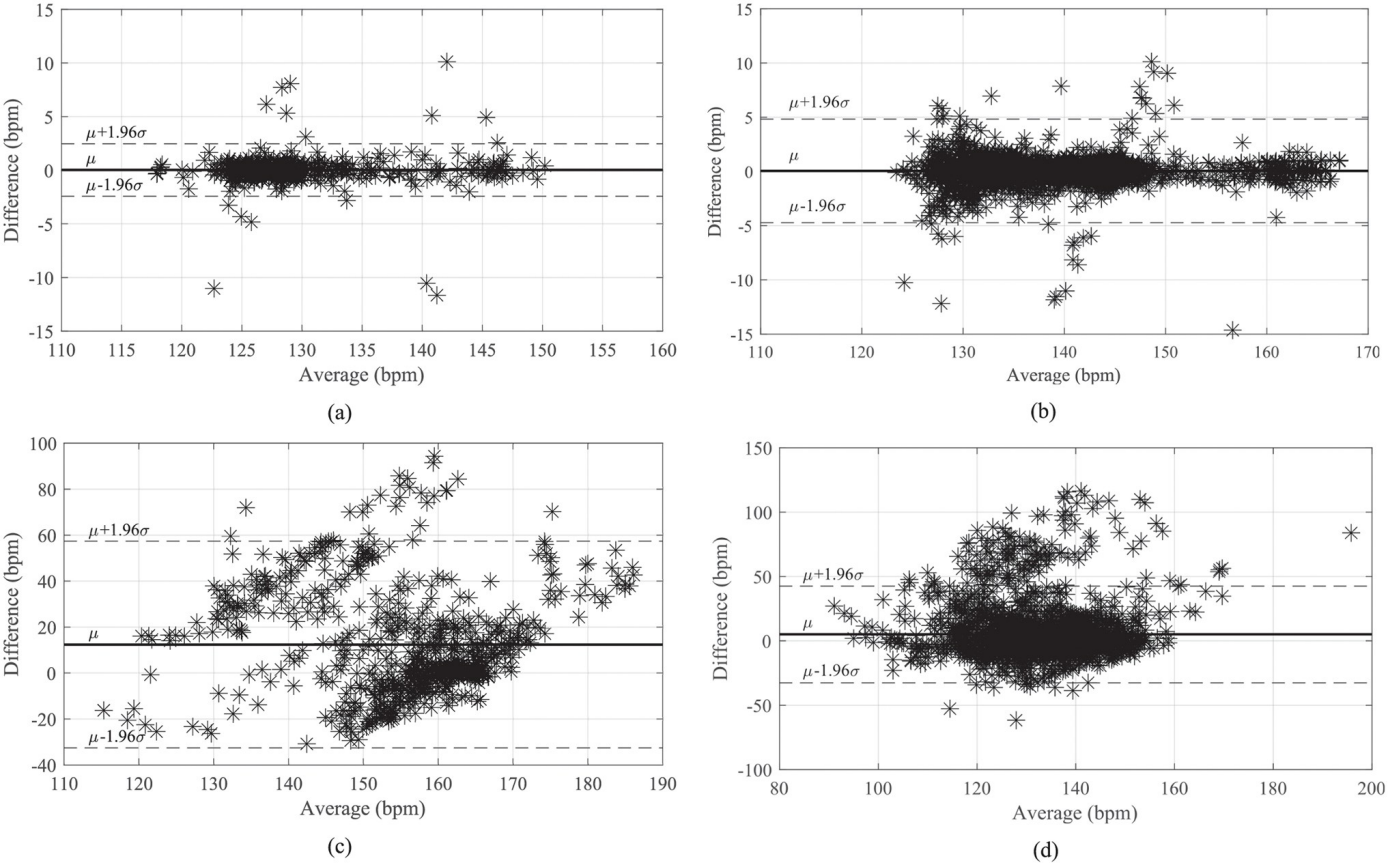
Example of Bland-Altman plot on good and bad results from both datasets for the FastICA-FTF method. (a) Labour dataset record r1, (b) Labour dataset record r3, (c) Pregnancy dataset record r4 and Pregnancy dataset record r10.

As part of the statistical analysis, we created the Bland-Altman plots, where two measurement results are compared. In our case, it is a comparison of the estimated fHR trace against the reference fHR trace. Herein, we present examples for the FastICA-FTF method, where Fig 6 shows examples of the Bland-Altman plot for good Fig 6a and 6c and bad result Fig 6b and 6d on both datasets. From the images Fig 6a and 6c, it can be seen that for good results, the line indicating the average value of *μ* was practically at 0 and there was a low value of 1.96*σ*, which indicates the minimum difference between the reference and filtered fHR curves. For bad results in Fig 6b and 6d, on the other hand, one can see a large shift of the line indicating the average value of *μ* deviating from the value 0 and a high value of 1.96*σ* indicating a large difference between the reference and filtered fHR trace. For a better overview, the *μ* and 1.96*σ* values obtained for FastICA-FTF method on all data (all records from both datasets) can be seen in Table 10.

**Table 10:** Mean values *μ* and values of 1.96*σ* determined for all records from both datasets tested for the FastICA-FTF method.

Record	Labour dataset		Pregnancy dataset	
	*μ*	1.96*σ*	*μ*	1.96*σ*
r1	0.02	2.44	0.07	6.84
r2	-0.30	10.70	1.34	17.94
r3	12.32	44.97	-0.39	8.20
r4	-1.21	12.34	0.05	4.77
r5	0.01	5.26	0.02	2.41
r6	-0.14	5.75	1.87	25.41
r7	0.83	13.50	0.61	18.97
r8	0.01	2.20	0.37	18.83
r9	-0.74	9.09	-0.05	7.18
r10	0.01	8.06	5.04	37.54
r11	-0.02	4.47	–	–
r12	0.02	1.49	–	–

### Analysis of the results

The results presented in the previous section showed that ICA-based methods combined with an adaptive FTF algorithm are able to effectively extract non-invasively sensed fECG. However, there are visible differences in accuracy when detecting fQRS complexes, namely 1) between individual ICA-based methods, 2) between individual recordings, and 3) between mean results of both datasets.

**Fig 7:**
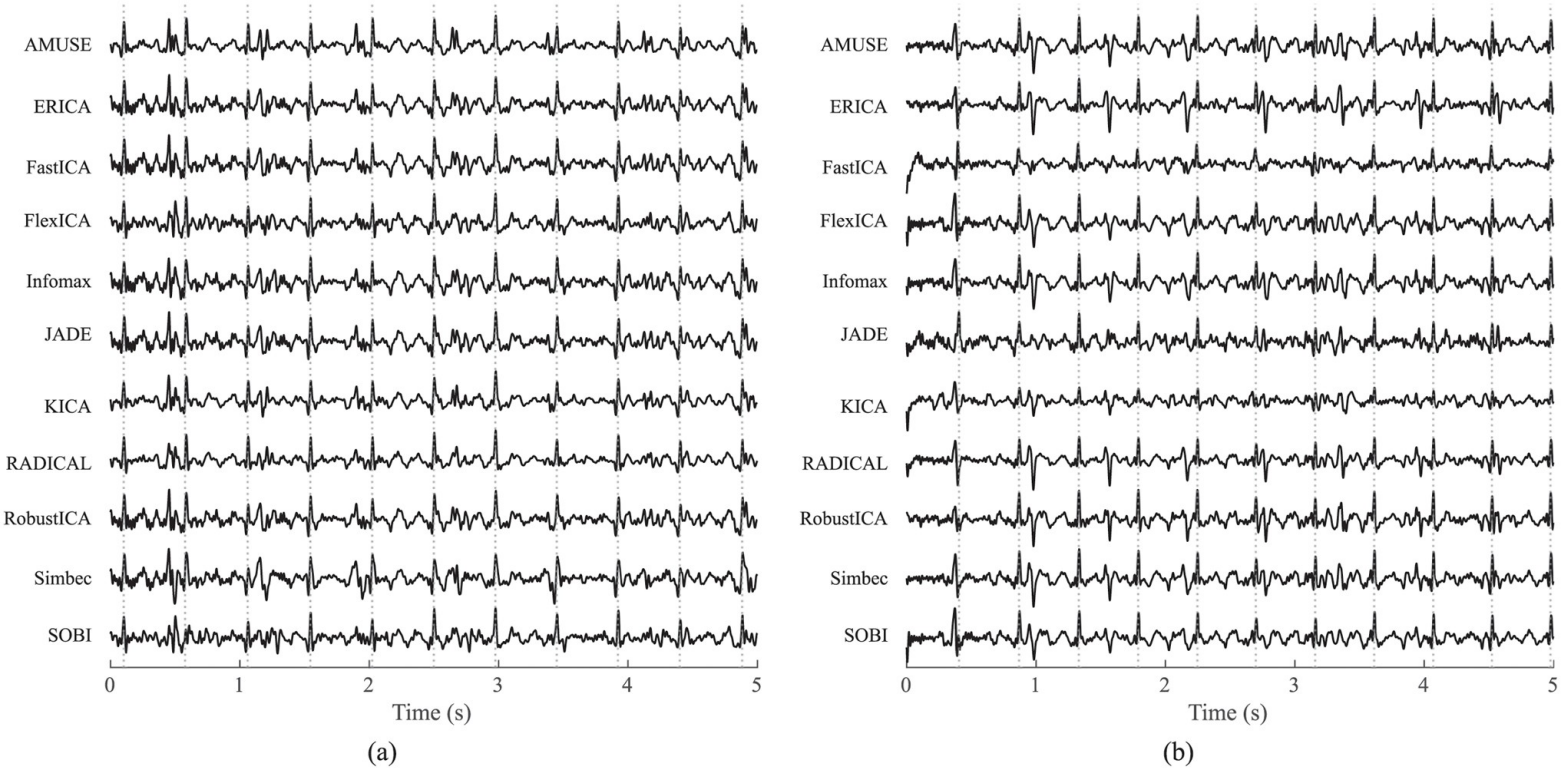
Example of waveforms of fECG signals extracted by all 11 tested ICA-based methods and the follow-up FTF applications (grey dashed lines represent annotations). (a) extracted signals from recording r7 of the Labour dataset and (b) extracted signals from recording r4 of the Pregnancy dataset.

**Fig 8:**
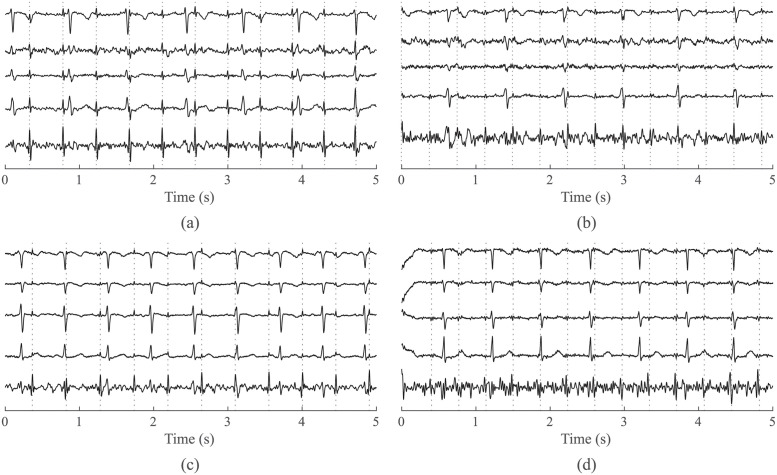
Example of effect of aECG input signal quality (always the four upper signals) on the resulting fECG extraction (always the last lower signal), where grey dashed lines represent annotations. (a) Labour dataset (r5); RADICAL-FTF; high-quality, (b) Labour dataset (r3); RADICAL-FTF; low-quality, (c) Pregnancy dataset (r3); FastICA-FTF; high-quality and (d) Pregnancy dataset (r7); FastICA-FTF; low-quality.

1. *Influence of algorithm selection*—the results of the statistical analysis, showed no statistically significant difference between the individual algorithms in terms of the accuracy they provide, only in the time needed for their calculations. However, there are visible differences between individual algorithms in some records. To show why each algorithm achieved different results with the same recording, we decided to compare the waveforms of the extracted fECG signals. An example of extracted signals for recording r7 of the Labour dataset is shown in Fig 7a. The recording shows that most methods struggled with suppressing the mQRS complex residues, leading to false positive detections. The maternal component was best filtered with the RADICAL method, which achieved the best performance (ACC = 95.07%). AMUSE and KICA were also able to eliminate the mQRS complexes well. However, along with the maternal component, they slightly suppressed the fQRS complexes as well, which lead to less accurate extraction (ACC = 87% and ACC = 82.16%, respectively). The SIMBEC method was the least successful of all methods in suppressing the mQRS complexes. In addition, the fetal component was reaching low levels compared to the maternal one, which led to low accuracy of fQRS complex detection (ACC = 69.01%).
  Nevertheless, in the case of recording r4 in the Pregnancy dataset (see Fig 5b), mQRS complexes were sufficiently suppressed by all methods, which led to effective extraction (ACC > 93%) by all methods. Visual comparison shows that the mQRS complex residues were best suppressed by the FastICA method, which had no substantial effect on the resulting extraction accuracy. The least accurate methods with this recording were RobustICA (ACC = 93.85%) and KICA (ACC = 94.16%), which failed to eliminate some of the residues of maternal components and the amplitude of some of the fQRS complexes were lower compared to the mQRS complex amplitude.
  It should be noted that the low average accuracy of the RADICAL method on the Pregnancy dataset is caused mainly due to one extremely low outcome on record r6, where the dropped below 20% (ACC = 14.47%) and further low accuracy outcome of the record r8 (ACC = 44.56%). On the other hand, for the other records, this method worked similarly as the other algorithms. We can therefore conclude that the RADICAL method was the least robust algorithm in the experiments on low-quality data.
  Finally, as proved by the results of the statistical analysis, the choice of the algorithm does not significantly influence the results. However, what really counts is the setting of the algorithms used. For ICA methods, the key parameters to be set are the number of components, convergence criterion, number of iterations and so on (depending on the type of ICA-based method). For adaptive algorithms, these are mainly the filter order, convergence constant or forgetting factor [15, 16]. We have found advantageous to use the optimization algorithms to find the optimal parameter setting instead of its manual selection in our previous studies [42, 43]. This allows the algorithm to appropriately adjust the different parts of the hybrid system according to the week of gestation, the position of the fetus and other circumstances.
2. *Influence of input signal quality*—the differences in extraction accuracy with each recording is caused primarily by the quality of aECG input signals. Earlier studies [44] already noted the dependency of proper positioning of sensing electrodes, quality of the acquired aECG signals, and the final quality of fECG extraction. Recordings, where the achieved accuracy of fQRS complex detection was low, contained aECG input signals with substandard quality. This means that the fetal component level compared to the maternal one was very low, in some cases even invisible, and some of the signals contained noise. Effective extraction is almost impossible with such signals, and greater attention should be paid to the proper positioning of the sensing electrodes and the measurement system setting when acquiring these signals. An example of the effect of aECG signal quality on the quality of the extracted fECG signal is shown in Fig 8. Example Fig 8a and 8c represent high-quality aECG signals from recording r5 of the Labour dataset and recording r3 of the Pregnancy dataset, respectively. In both cases, the fetal component level is high enough with respect to the maternal one and the signals are not burdened with other interference. The extraction using these aECG signals was very accurate (ACC = 97.01% and ACC = 93.82%, respectively), as mECG was sufficiently suppressed and the fECG was effectively enhanced. In contrast, example Fig 8b and 8d present low-quality aECG signals from recording r3 of the Labour dataset and recording r7 of the Pregnancy dataset, respectively. The fetal component of the aECG signals is barely visible in these recordings, and some of the signals are burdened with interference. This has led to insufficiently accurate fECG extraction (ACC = 47.91% and ACC = 79.16%, respectively).
3. *Influence of dataset used*—comparison of the results of both datasets show that the mean accuracy achieved during fQRS complex detection in recordings from the Labour dataset was higher than in case of the Pregnancy dataset recordings (ACC = 84.14%). Based on the information from [38], in which the authors described and analyzed the datasets, along with our own findings, such difference could be caused by the following:
  - The first obvious reason for such differences is that in Labour dataset, the gestation age of the fetuses monitored was higher (38–42 weeks) in comparison to those in Pregnancy dataset (32–42 weeks), which generally means that the amplitude of the fetal ECG is higher and thus easier to extract [1, 45–48]. This is confirmed in the Labour dataset, where according to the information from [38], the mean mQRS:fQRS complex amplitude ratio was 2, while the ratio in Pregnancy dataset was about 3.5. A conclusion can be drawn from this that with lesser amplitude difference between the maternal and fetal component in Labour dataset, the algorithms were better able to suppress the maternal component and extract higher-quality fECG.
  - In [38], the authors determined parameters describing changes to fQRS complex amplitudes. In the case of Labour dataset, amplitude levels were more stable compared to fQRS complex amplitudes in the Pregnancy dataset. Lower variance of fQRS complex amplitudes therefore allows acquiring more uniform detection function, which then leads to more accurate detection in the Labour dataset recordings.

## Summary and discussion

Since not all ICA-based methods worked well for various signals, we decided to create a summarizing evaluation of the algorithms, see Table 11. The goal was to find which algorithm generally detects the most TP values and least FP and FN values, while achieving the highest mean values of ACC, SE, PPV, and F1 in both datasets. The highest number of correctly detected fQRS complexes, i.e. TP values, were acquired with the FastICA method. In addition, the FastICA method detected the lowest number of FR and FN values, which led to the most accurate detection of fQRS complexes according to all the applied parameters—ACC, SE, PPV and F1. Conversely, the lowest number of TP values and the highest number of FP and FN values were detected by the SOBI method. This method can therefore be considered as the least suitable for extracting non-invasive fECG.

**Table 11:** Summary of results based on a sum of all TP, FP, and FN values and mean values of ACC, SE, PPV, and F1 statistic parameters acquired from both, labour and pregnancy dataset (the best results are bold).

Algorithm	∑TP	∑FP	∑FN	øACC (%)	øSE (%)	øPPV (%)	øF1 (%)
AMUSE	31625	5229	4553	76.38	87.42	85.81	86.61
ERICA	32438	4621	3740	79.51	89.66	87.53	88.58
FastICA	**33332**	**3636**	**2846**	**83.72**	**92.13**	**90.16**	**91.14**
FlexICA	31781	5459	4397	76.33	87.85	85.34	86.58
Infomax	31556	5034	4622	76.57	87.22	86.24	86.73
JADE	32546	4543	3632	79.92	89.96	87.75	88.84
KICA	32861	4181	3317	81.42	90.83	88.71	89.76
RADICAL	31485	3988	4693	78.39	87.03	88.76	87.88
RobustICA	32239	4687	3939	78.89	89.11	87.31	88.20
SIMBEC	31977	5165	4201	77.35	88.39	86.09	87.23
SOBI	31301	5874	4877	74.43	86.52	84.20	85.34

Table 12 provides the comparison of all tested ICA-based methods. For all tested algorithms, we provide their strengths and limitations in the first two columns of this table. The other columns indicate the order of the methods according to the average results achieved in this experiment for all the tested parameters for both datasets. The last column corresponds to the sum of the placements. The results of this comparison showed that the FastICA algorithm seems to be the best compromise from the ICA-based methods, when considering the extraction accuracy and computation speed. Also, the algorithm’s strengths are numerous in comparison with the other methods. The disadvantage posed by the FastICA is that it changes the order of extracted components (aECG*, mECG, and noise) and signal amplitude. Therefore, their use the in automated devices would require development of a precise algorithm for automatic identification of the components.

**Table 12:** Comparison of ICA-based methods consistent with the results and own experience. Numbers in each column indicate the order of effectiveness of the method according to the given parameter.

Algorithm	Strength	Limitation	ACC	SE	PPV	F1	Speed	Total
AMUSE	Fast Easy to implement	Sensitive to low-quality signals	9	8	9	9	2	37
ERICA	Extracts quality mECG	Random output amplitude	4	4	5	4	9	26
FastICA	Fast Extracts quality mECG Robust to low-quality signals Arbitrary number of ICs	Random output components Random output amplitude Needs a priori knowledge	1	1	1	1	4	8
FlexICA	Arbitrary number of ICs	Random output amplitude Needs a priori knowledge	10	7	10	10	8	45
Infomax	Extracts quality mECG Arbitrary number of ICs	Slow Random output amplitude Needs a priori knowledge	8	9	7	8	11	43
JADE	Fast Extracts quality mECG	Random output amplitude	3	3	4	3	3	16
KICA	Fast Easy to implement Robust to low-quality signals	Random output amplitude	2	2	3	2	1	10
RADICAL	Extracts quality mECG	Slow Random output amplitude Sensitive to low-quality signals	6	10	2	6	10	34
RobustICA	Fast Extracts quality mECG	Random output amplitude Sensitive to low-quality signals Needs a priori knowledge	5	5	6	5	5	26
Simbec	Fast Extracts quality mECG Arbitrary number of ICs	Random output amplitude	7	6	8	7	6	34
SOBI	Easy to implement	Random output amplitude Needs a priori knowledge	11	11	11	11	7	51

Another problem may be the randomness effect, which causes different output components each time the ICA-based method is run. This means that an ICA-based method would have to be run several times to eliminate this randomness effect. The data used in this study was of good/medium quality, so this randomness effect was minimal, which is why we neglected it after the initial experiments. In these initial experiments, we tried to run individual algorithms repeatedly on the same recording and the same combinations of electrodes, and the randomness effect did not manifest itself significantly. In the case of low-quality signals, of course, the efficiency of the algorithms would drop sharply and the randomness effect could have a big influence. With low quality signals, where the fECG is almost invisible, it may even happen that the ICA-based method will not be able to extract the necessary output components at all, even with repeated execution. The problem of randomness effect was for example addressed in a study [49]. The future study would have to be carried out on a different dataset which would include more of a low-quality data where the randomness effect would manifest itself. This could mainly be the relatively new NInFEA dataset [50], where reference annotations are not yet available. This was crucial for our study and therefore we did not include this dataset herein. In addition, the PhysioNet Challenge 2013 dataset [51] could theoretically be used as it does contain reference annotations, but here we encountered the problem that part of the dataset is synthetic and it is not clearly noted which one.

In order to make the fQRS complex detection even more accurate, post-processing algorithms (e.g. WT or EMD) could be tested in the future as they might improve the extraction quality. The future research should also focus on testing the proposed algorithm from the perspective of morphological analysis feasibility (such as ST segment analysis or QT interval analysis), which is very important for clinical practice. The ST segment analysis is a particularly important indicator of fetus’ health condition and can be used to detect fetal hypoxia very accurately (more accurately than with conventional CTG). The robustness of the algorithm should be determined by means of testing pathological recordings and abnormality recordings in addition to physiology recordings.

## Conclusion

This study provided a comparison of available ICA algorithms for the fECG signal processing. The results on two tested datasets, Pregnancy dataset and Labour dataset, showed superior results of the FastICA algorithm and the follow-up FTF application in terms of accuracy (ACC = 83.72%, SE = 92.13%, PPV = 90.16%, F1 = 91.14%). Moreover, the computation time needed was comparable with the other tested methods (mean of 0.452 s), allowing the algorithm implementation for the real-time applications. These results provide a significant first step towards creating a suitable hybrid method for NI-fECG extraction.

## Supporting information

S1 File Used datasets. (ZIP)

S2 File Extracted signals from labour dataset. (ZIP)

S3 File Extracted signals from pregnancy dataset. (ZIP)
